# Commentary: Constructing nonhuman animal emotion

**DOI:** 10.3389/fpsyg.2017.02070

**Published:** 2017-11-28

**Authors:** Marco Viola

**Affiliations:** ^1^Center for Neurocognition, Epistemology and Theoretical Syntax, Istituto Universitario di Studi Superiori di Pavia (IUSS), Pavia, Italy; ^2^Vita-Salute San Raffaele University, Milan, Italy

**Keywords:** emotions, basic emotions, constructed emotions, animal emotions, affect, core affect

In a recent opinion paper, Bliss-Moreau argues for a new framework for studying non-human animal emotions. Contrary to Classical Views of Emotion (CVE) such as Basic Emotion theories, she claims that “emotions are not modules or hardwired circuits, but rather emerge from a combination of ingredients” (Bliss-Moreau, 2017, p. 185), as predicted by Theories of Constructed Emotions (TCE). Such an endeavor is interesting and praiseworthy, as it promises to overcome the anthropomorphic vice to impose human emotion categories on the whole animal kingdom. Inasmuch as it makes room for the construction of genuinely non-human emotions, TCE allegedly enables us to “understand animal minds for their own sake” (Barrett, 2017, p. 276). However, I suspect that this enterprise rests on shaky foundations.

TCE proponents (most notably Barrett, 2006) contend the central assumption of the CVE, i.e., that emotion categories such as “fear” and “anger” each denote a biologically inherited mechanism which is shared by all humankind, and arguably by several mammals. Behavioral and neuroanatomical homologies in mammals such as those described by Panksepp (e.g., Panksepp, 2007) constitute *prima facie* counterevidence to their view. Barrett et al. (2007) concede that these count as similarities in *behaviors* (and in behavioral circuits). Nonetheless, they claim that inferring an *emotion* from a behavior would be a mistake, which Barrett (2017, p. 272) dubs the *mental inference fallacy*.

One thing to keep in mind here is that different scholars seem to have different explanatory targets when they speak of emotion and folk emotion terms (e.g., “fear”). While some think of them as functional states mainly defined by their behavioral outcomes (in behavioristic-flavored *third-person* perspective; e.g., Panksepp, 2007; Adolphs, 2017), others save these folk terms to indicate phenomenological states (assessable through a *first-person* perspective; e.g., LeDoux, 2012; Barrett, 2017). Inasmuch TCE advocates adhere to this second usage of emotion terms (which is likely also closer to their vernacular meaning), they have good reasons for casting doubts upon mental inferences from behaviors to emotions, since according to their jargon that entails inferring first-person states from third-person observation.

TCE's critique against CVE sounds quite convincing (though not conclusive; see Scarantino, 2015; Celeghin et al., 2017). Unfortunately, I find their counterproposal far less convincing. Indeed, at the present stage the model they propose might be equally if not more flawed than CVE's.

Varieties of TCE differ in what ingredients are posited for emotional recipes, and in how important they are, e.g., semantic knowledge, past experiences, social roles, and norms. However, “at the core of [every] TCE is “affect”—a global state characterized by valence and arousal that forms the basis of emotions” (Bliss-Moreau, 2017, p. 185). Whilst not *sufficient*, affect is a *necessary* ingredient for emotion.

Both Bliss-Moreau and her mentor Barrett take for granted that non-human animals experience affect. They also seem committed to the view that affect is best described by some degree of arousal and valence (Barrett and Bliss-Moreau, 2009). Having asked whether animals experience affect, Barrett answers that “we can give a pretty confident yes, based on some biological and behavioral clues” (Barrett, 2017, p. 268). However, I am afraid that the available clues suggest otherwise.

Several neuroimaging studies in human sought to map either discrete emotion categories or affective properties (valence, arousal) onto some neural basis. Meta-anlyses reveal that the selectivity of valence/arousal is as scarce as that of the discrete emotion categories (Wager et al., 2015), and perhaps even worse (Kragel and LaBar, 2015). But then, if valence and arousal are not even firmly grounded in human neurobiology, why should they be a common properties of the whole animal kingdom?

Behavioral evidence is likewise controversial. According to Bliss-Moreau (2017, 185), “affect is likely present in most non-plant organisms, although in those lacking nervous systems or with simple nervous systems […] it may appear in rudimentary form. For example, bacteria move toward positive things (e.g., food) and away from negative ones (e.g., acid), indicating that they can use signals about affective value to guide behavior.” Due to the brevity of her article, she does not elaborate further. Instead, she refers to (LeDoux, 2012) for a discussion of this point. However, LeDoux's paper hardly supports such a claim. Tackling the conceptual confusion surrounding the first-person versus third–person meanings of “emotion” and emotion terms, LeDoux propose to save them for the first-person states, and dubs the homological structures that Panksepp had in mind (and that are responsible for homolog behaviors) “survival circuits.” He claims that we can and should seek for homology in survival circuits (starting with bacteria), and that we should refrain from assigning phenomenal states to non-human organisms: “survival circuits have their ultimate origins in primordial mechanisms that were present in early life forms. This is suggested by the fact that extant single-cell organisms, such as bacteria, have the capacity to retract from harmful chemicals and to accept chemicals that have nutritional value” (LeDoux, 2012, p. 655). Thus, *based on behavioral similarities*, what he hypothesizes we might share with bacteria and other living beings (especially mammals) is some kind of survival circuits, *not a first-person affective state characterized by valence and arousal*.

What is at stake here is not just an infelicitous reference. Indeed, valence and arousal are meant to describe felt experience in human, and their validation in human heavily relies on first-person data, e.g., verbal judgments and self-reports (Russell, 1980). But I can see no way to gather first-person data from non-human animals: all we have left are thus third-person data. But then, *projecting our human first-person affective structure to non-human animals based on third-person data seems no less a mental inference fallacy then projecting the first-person states correlated with our discrete categories*. And given that arousal/valence are admittedly only “descriptive features of core affect that bear no resemblance to or inform about how affect is caused” (Barrett and Bliss-Moreau, 2009, p. 188), discrete categories seem at least suited for grasping causal processes underlying behavioral homologies (Panksepp, 2007; LeDoux, 2012). In the light of this, the bargain proposed by TCE on non-human animal emotion hardly seems a good deal.

## Author contributions

The author confirms being the sole contributor of this work and approved it for publication.

### Conflict of interest statement

The author declares that the research was conducted in the absence of any commercial or financial relationships that could be construed as a potential conflict of interest.
